# Carbon Nanotube Migration in Melt-Compounded PEO/PE Blends and Its Impact on Electrical and Rheological Properties

**DOI:** 10.3390/nano12213772

**Published:** 2022-10-26

**Authors:** Calin Constantin Lencar, Shashank Ramakrishnan, Uttandaraman Sundararaj

**Affiliations:** Department of Chemical and Petroleum Engineering, University of Calgary, 2500 University Drive NW, Calgary, AB T2N 1N4, Canada

**Keywords:** polymer nanocomposite, electrical conductivity, mixing, polymer blends, electromagnetic interference shielding, carbon nanotube, filler migration, polyethylene

## Abstract

In this work, the effects of MWCNT concentration and mixing time on the migration of multi-walled carbon nanotubes (MWCNTs) within polyethylene oxide (PEO)/polyethylene (PE) blends are studied. Two-step mixing used to pre-localize MWCNTs within the PE phase and subsequently to observe their migration into the thermodynamically favored PEO phase. SEM micrographs show that many MWCNTs migrated into PEO. PEO/PE 40:60 polymer blend nanocomposites with 3 vol% MWCNTs mixed for short durations exhibited exceptional electromagnetic interference shielding effectiveness (EMI SE) and electrical conductivity (14.1 dB and 22.1 S/m, respectively), with properties dropping significantly at higher mixing times, suggesting the disruption of percolated MWCNT networks within the PE phase. PE grafted with maleic anhydride (PEMA) was introduced as a compatibilizer to arrest the migration of MWCNTs by creating a barrier at the PEO/PE interface. For the compatibilized system, EMI SE and electrical conductivity measurements showed a peak in electrical properties at 5 min of mixing (15.6 dB and 68.7 S/m), higher than those found for uncompatibilized systems. These improvements suggest that compatibilization can be effective at halting MWCNT migration. Although utilizing differences in thermodynamic affinity to draw MWCNTs toward the polymer/polymer interface of polymer blend systems can be an effective way to achieve interfacial localization, an excessively low viscosity of the destination phase may play a major role in reducing the entrapment of MWCNTs at the interface.

## 1. Introduction

Electronic devices have made the world more connected than ever. Smart phones, laptops, and other such devices have made the internet more accessible, and internet of things have connected everything in our daily lives to our devices. The utility of smart devices has fueled huge growth in their global demand. This growing demand has led to an explosion in wireless data traffic [1]. Unfortunately, all electronic devices (especially wireless devices) emit electromagnetic (EM) waves as part of their regular operation. These EM waves can interfere with other critical devices, including medical equipment, navigational equipment, and communications devices [2,3,4]. This interference can lead to faulty operation or even total failure of the affected equipment. Furthermore, there are health implications due to high exposure to EM waves, and serious chronic health issues may arise in humans [5,6]. Due to the hazards of electromagnetic interference (EMI), the demand for proper shielding measures has grown significantly. Historically, metals have been utilized to shield against EMI, but they are expensive, difficult to shape, and susceptible to corrosion [7,8]. Furthermore, metals protect against incident EM waves by reflecting them back into the environment, and these reflected waves can still interfere with other devices [9]. Polymer, ceramic, and metal-based nanocomposites offer numerous advantages, including their light weight, resistance to corrosion, and high tuneability. Metal matrix nanocomposites utilize the exceptional electrical properties offered by metals and seek to combat the low mechanical yield of metals via nano-reinforcement [10]. Other metal-based nanocomposites, such as those prepared by Ji et al., utilize open-celled foams containing metal nanowires grafted on carbon nanotubes (CNTs) to shield against incident EMI [11]. Ceramic nanocomposites containing nanoparticles such as CNTs negate the typically high brittleness seen in ceramics and offer unique opportunities for multifaceted applications due to the high electrical properties of CNTs [12]. Polymer nanocomposites (PNCs) containing multi-walled carbon nanotubes (MWCNTs) are especially interesting, due to their high impact properties and processability [13]. Additionally, PNCs containing MWCNTs primarily attenuate incident EMI via absorption and this can significantly reduce EMI smog [14,15,16]. MWCNTs possess superb conductivity, tensile strength, and a high aspect ratio, which makes them ideal for imparting electrical properties to polymers through interconnected conductive networks [17,18]. Single walled carbon nanotubes (SWCNTs) can also be utilized, but their application is often inhibited by high synthesis costs. Several works have achieved exceptional electrical properties and thermal stability of SWCNTs via polybenzoxazines and oxadiazole-linked conjugated microporous polymers, but these processes have limited scalability for industrial applications [19,20].

Previous works have shown that polymer blend nanocomposites (PBNs) containing MWCNTs can be used to develop inexpensive conductive materials for advanced applications, including in the medical and aerospace sectors. Sumita et al. [21] were the first to show that by localizing carbon black (CB) within one phase of HDPE/PP and PP/PMMA PBN systems, the effective local concentration of CB in one phase could be increased, thus reducing the total quantity of conductive filler required to form a percolated network within the blend, in a phenomenon often dubbed “double percolation”. Double percolation has also been studied in various PBN systems containing MWCNTs [22,23,24]. In these systems, it is important that the polymer blend has a co-continuous morphology, so that the percolation of nanofiller within one of the phases leads to the formation of a continuous nanofiller network. Recent work has shown that the concept of double percolation of MWCNTs within polymer blends can be taken further, by locating them at the polymer/polymer interface. Zhang et al. [25] used amine functionalized MWCNTs in blends of PA6/PVDF to achieve interfacial localization, resulting significantly reduced percolation thresholds. Wu et al. [26] made use of carboxylic-functionalized MWCNTs within blends of poly (ε-caprolactone)/polylactide (PCL/PLA) with similar effect. Solution mixing techniques have also been adopted to achieve interfacial localization of MWCNTs within polymer blends [27,28]. Unfortunately, the use of solution mixing strategies or functionalized MWCNTs increases the cost of preparing PBN materials, hindering the scalability of these systems for commercial purposes.

Understanding how MWCNTs move within a given polymer blend is an important first step to preparing PBN systems with MWCNTs localized at the polymer/polymer interface. MWCNTs should initially be in the phase with lower thermodynamic affinity to encourage their migration toward the interface with their preferred phase. Young’s equation is often used to predict the thermodynamic preference of MWCNTs within binary polymer blends [21]:

$$\omega_{A/B}=\frac{\sigma_{A/\mathrm{MWCNT}}-\sigma_{\mathrm{MWCNT}/B}}{\sigma_{A/B}}$$

where

$\omega_{A/B}$—wettability of MWCNTs within a blend of polymer A and B,

$\sigma_{A/\mathrm{MWCNT}}$—surface energy between MWCNTs and polymer A,

$\sigma_{\mathrm{MWCNT}/B}$—surface energy between the MWCNTs and polymer B, and

$\sigma_{A/B}$—surface energy between polymers A and B.

When $\omega_{A/B}>1$, MWCNTs will prefer polymer B. When $\omega_{A/B}<-1$, MWCNTs will prefer polymer A. Finally, when $-1<\omega_{A/B}<1$, MWCNTs will tend to settle at the polymer A/polymer B interface.

In our previous work [29], poly (vinylidene difluoride) (PVDF)/polyethylene (PE) blends of varying blend ratios containing 2 vol% MWCNTs were studied to observe the effect of blend morphology and mixing time on the migration behavior of MWCNTs during melt mixing. MWCNTs were initially localized within PE and migrated towards PVDF during subsequent melt-mixing. Although MWCNTs thermodynamically favor PVDF (based on Young’s equation), the higher viscosity of PVDF relative to PE was expected to retard the migration of MWCNTs from PE into PVDF when MWCNTs reached the interface. SEM images both confirmed that MWCNTs migrated toward the PVDF/PE interface and became trapped there. A modified version of Göldel et al.’s [30] “Slim-Fast” mechanism was used to conceptualize the migration behavior of MWCNTs within the PVDF/PE blend. Short, straight MWCNTs are more likely to penetrate the interface while coiled MWCNTs and MWCNT agglomerates are more likely to become trapped at the polymer/polymer interface.

The objective of the current work is to study the phase migration of MWCNTs within co-continuous blends of polyethylene oxide (PEO) and PE, with the aim of producing MWCNT-based PBN materials with exceptional electrical properties at low MWCNT concentrations. The concentration of MWCNTs within the PE phase at the start of mixing with PEO was varied to study the difference in migration behavior of individual and agglomerated MWCNTs. MWCNTs were selected for their superb electrical and mechanical properties, high aspect ratio, and low-cost relative to nanoparticles, such as single-walled carbon nanotubes (SWCNTs) or graphene [31]. PEO was chosen because it has good affinity for MWCNTs compared to PE, it has low viscosity, and its water solubility allows it to be easily extracted from blends with PE [32]. The same PE was used in this work as in our previous work with PVDF [29] to provide a direct comparison between the two systems. A low-cost polymer with exceptional impact properties and high processability, PE is an excellent choice for commercial PBN systems. The purpose of using a PE with a relatively high viscosity versus PEO was to study the relative significance of nanoparticle geometry (i.e., individual MWCNTs vs. agglomerated MWCNTs) and polymer viscosity on MWCNT phase migration. Better understanding of the migration behavior of MWCNTs allows us to better design high performance MWCNT-based materials for commercial applications.

## 2. Materials and Methods

### 2.1. Materials and Sample Preparation

PBN samples were prepared using PEO (Polyox^TM^ WSR N10) supplied by DuPont (Wilmington, DE, USA), PE (Lumicene^TM^ M3581 uv) supplied by Total SA (Houston, TX, USA), PE grafted with maleic anhydride (PEMA) (OREVAC^®^ 18340) supplied by Arkema S.A. (Colombes, France), and MWCNTs (NC7000) supplied by Nanocyl S.A. (Sambreville, Belgium). Based on the specifications provided by the manufacturer, NC7000 has an average diameter of 9.5 nm, an average length of 1.5 µm, and aspect ratio of 158, 90% purity and an electrical conductivity of approximately 10^6^ S/m. PEO and PE were vacuum dried at 60 °C for 24 h before use.

**Figure 1:**
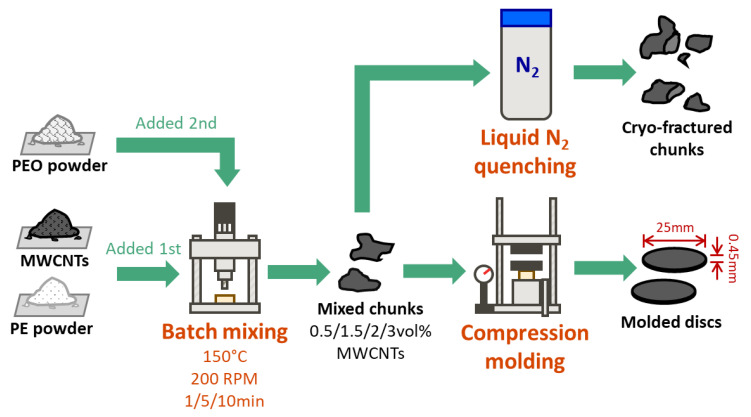
Schematic representation of procedure used to prepare the PEO/PE/MWCNT nanocomposite samples studied in this work.

PBN samples with a PEO/PE ratio of 40:60 were prepared at MWCNT concentrations of 0.5, 1.5, 2, and 3 vol% using two-step mixing. MWCNT powder and PE powder were dry mixed and added to the mixing cup of an Alberta Polymer Asymmetric Mini-mixer (APAM) (University of Calgary, Calgary, AB, Canada) and left to melt for 2 min without rotation [33]. The mixture was then melt-compounded at 200 rpm for 5 min to create a well-mixed PE/MWCNT composite. Mixing was then halted, and PEO powder was introduced to the mixing cup, and left to melt for 2 min without rotation. Finally, the mixture was melt-compounded at 200 rpm for an additional 1, 5, and 10 min. All melting and mixing steps in the APAM were done at a constant temperature of 150 °C. Samples were rapidly removed at the end of the final blending step and chunks of the sample were quenched in liquid nitrogen to freeze the sample morphology. The rest of the sample was molded into circular discs (diameter = 25 mm, thickness = 0.45 mm) using a Carver compression molder (model 3912) (Carver Inc., Wabash, IN, USA) at 150 °C and 35 MPa for 10 min. A minimum of four specimens were prepared for each sample to measure electrical conductivity, EMI shielding and rheological properties. An outline of the procedure for preparing PEO/PE/MWCNT samples can be found in Figure 1 below.

### 2.2. Sample Characterization

Cryo-fractured samples were mounted and imaged under a low vacuum using a Quanta FEG 250 VP-FESEM (variable pressure field emission SEM) (FEI Company, Hillsboro, OR, USA). A large field detector (LFD) was used to take secondary electron images to observe the detailed sample topography (including MWCNTs).

EMI shielding measurements were performed in the X-band frequency range (8.2–12.4 GHz) using a vector network analyzer (ENA Model E5071C) (Agilent Technologies, Santa Clara, CA, USA), with a connectedWR-90 rectangular waveguide. The X-band is a radar frequency usually used in civil and military applications, aircraft and sea craft detection and monitoring [34]. Although the X-band exists in a higher frequency range than the frequencies typically used by wireless smart devices, several works have shown that EMI shielding effectiveness increases with decreasing frequency [7]. This suggests that EMI shielding materials that perform well in the X-band will perform even better at lower frequencies. EMI SE values were derived from scattering parameters (see Supplementary Materials) based on measured data [7]. DC electrical conductivity of the samples was measured via a Loresta GP (model MCP-T610) resistivity meter (Mitsubishi Chemical Co., Tokyo, Japan), attached to an ESP probe. Measurements were performed on 4 specimens for each sample, with the average values of EMI SE and conductivity reported within this work.

Rheological tests were performed using an Anton-Paar rheometer (MCR 302) (Anton-Paar GmbH, Graz, Austria) with a 25 mm diameter parallel plate and a gap size of 0.45 mm. Linear frequency sweeps in the range of 600–0.1 rad/s were performed at a constant strain of 0.1% for 3 specimens. Frequency sweeps were followed by strain sweeps in the range of 0.1–1000% strain to confirm the linear viscoelastic region (LVR) of the specimens. All tests were conducted at a constant temperature of 150 °C.

## 3. Results

### 3.1. Theoretical Surface Energy Models

Prior to preparing the PEO/PE/MWCNT blend nanocomposites, a theoretical model based on a modified Young’s equation adapted by Sumita et al. [21] was used to predict the surface energies of the blend components within the system. The surface energy calculations were performed using surface energy values for the individual components reported in literature, obtained at 25 °C [35,36]. Details on the equations used to calculate the surface energies and wettability values can be found in the Supplemental Materials.

Based on the surface energy and wettability values in Table 1, MWCNTs will prefer to localize within PEO over PE. Consequently, MWCNTs were pre-localized within PE prior to subsequent melt-compounding with PEO to see if MWCNT would migrate from PE to the thermodynamically preferred PEO phase. For more details on the data used to calculate the parameter in Table 1, refer to Table S1 of the Supplementary Materials.

### 3.2. Imaging Results

**Figure 2:**
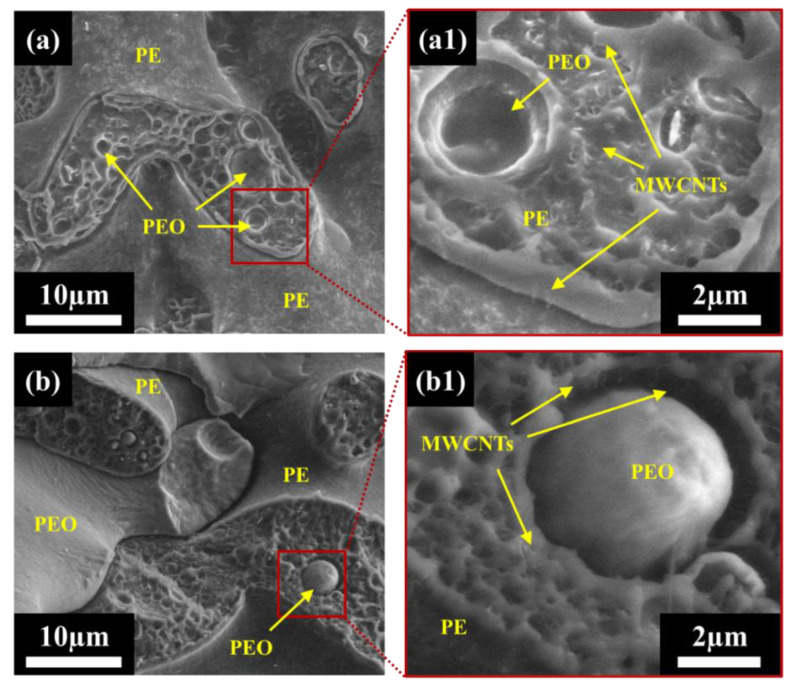
Low field detector (LFD) SEM images of PEO/PE 40:60 with 0.5 vol% MWCNTs mixed for (**a**) 1 min and (**b**) 10 min. Images (**a1**) and (**b1**) show PEO droplets at higher magnification.

Samples were initially studied using light microscopy (LM) imaging to better understand the dispersion and localization of MWCNTs within the PEO/PE blends, and the results can be found in Figure S1 of the Supplementary Materials. The detailed morphology of the prepared 40:60 blends systems were studied via SEM to better understand the morphology of the system, and to study the localization of MWCNTs within the blend system, especially at the PEO/PE interface. SEM images of pure PE/PEO blends without MWCNTs can be seen in Figure S2 of the Supplementary Materials. Figure 2 shows SEM micrographs of PEO/PE 40:60 blends containing 0.5 vol% MWCNTs mixed for 1 min and 10 min of mixing. At 1 min of mixing Figure 2a), the PE phase appears to have a grainy structure, which is due to the MWCNTs within the PE phase. In contrast, the PEO phase is not present within the micrograph, having fallen off the fracture surface due to poor adhesion to the PE phase. This poor adhesion suggests that MWCNTs have not yet migrated to the PEO/PE interface, which would improve interfacial adhesion. At higher magnification (Figure 2(a1)), individual MWCNTs appear as hairs along the surface of the PE phase. 

At 10 min of mixing (Figure 2b), PEO domains (which appear far smoother than the PE domains) can be seen along the sample surface. The PEO appears as large co-continuous domains with PE, and also as small droplets that are imbedded within PE. Droplets of PEO are likely sticking to the PE phase due to the presence of MWCNTs at the blend interface, i.e., MWCNT improves the interfacial adhesion via bridging. At higher magnification (Figure 2(b1)), individual MWCNTs can be seen along the PE surface, and PE ridges that are concentrated with MWCNTS can be seen surrounding PEO droplets. Thus, it is apparent that MWCNTs are migrating towards the PEO/PE interface and likely penetrating PEO.

**Figure 3:**
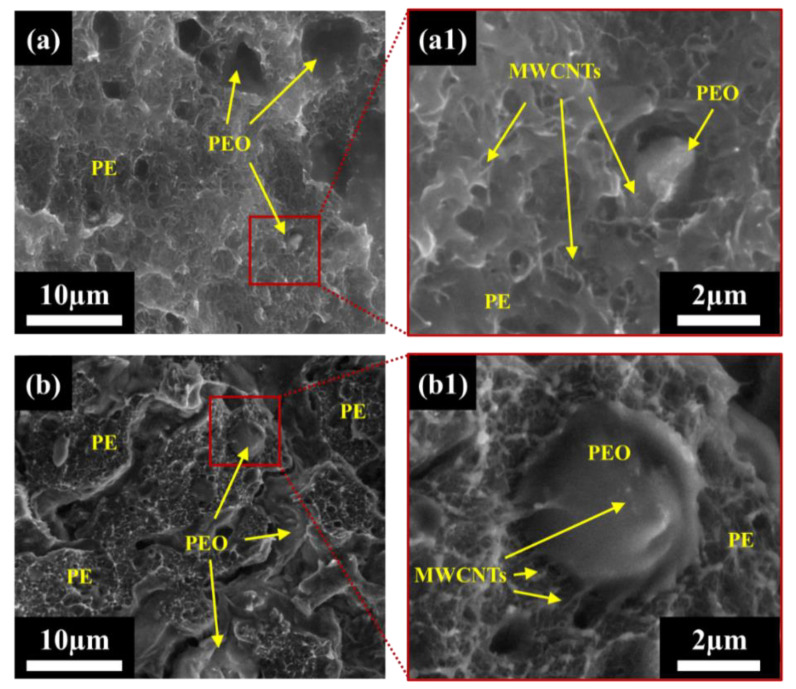
Low field detector (LFD) SEM images of PEO/PE 40:60 with 3 vol% MWCNTs mixed for (**a**) 1 min and (**b**) 10 min. Images (**a1**) and (**b1**) show PEO droplets at higher magnification.

Figure 3 shows SEM micrographs of PEO/PE 40:60 blends containing 3 vol% MWCNTs mixed for 1 min and 10 min. At 1 min of mixing (Figure 3a), the PE phase appears the have a very hair-like texture, due to the very high concentration of MWCNTs present therein. Similar to the 0.5 vol% blend at 1 min of mixing (Figure 3a or Figure 3(a1)), the PEO phase is seldom actually present since much of it has likely fallen from the fracture surface due to poor interfacial adhesion with PE. At higher magnifications (Figure 3(a1)), individual MWCNTs can clearly be seen along the surface of the PE phase, and smaller droplets of PEO that have been filled with MWCNTs can also be seen. At 10 min of mixing (Figure 3b), many domains of PEO (i.e., smaller PEO droplets and larger continuous PEO domains) can be observed. Once again, this is likely due to MWCNTs bridging the PEO/PE interface as they migrate into PEO. At higher magnification (Figure 3(b1)), dense networks of MWCNTs can be seen surrounding PEO droplets, and many MWCNTs can be seen straddling the PEO/PE, and even fully embedded within PEO.

### 3.3. Electrical Properties: DC Conductivity, EMI Shielding Effectiveness and Permittivity

**Figure 4:**
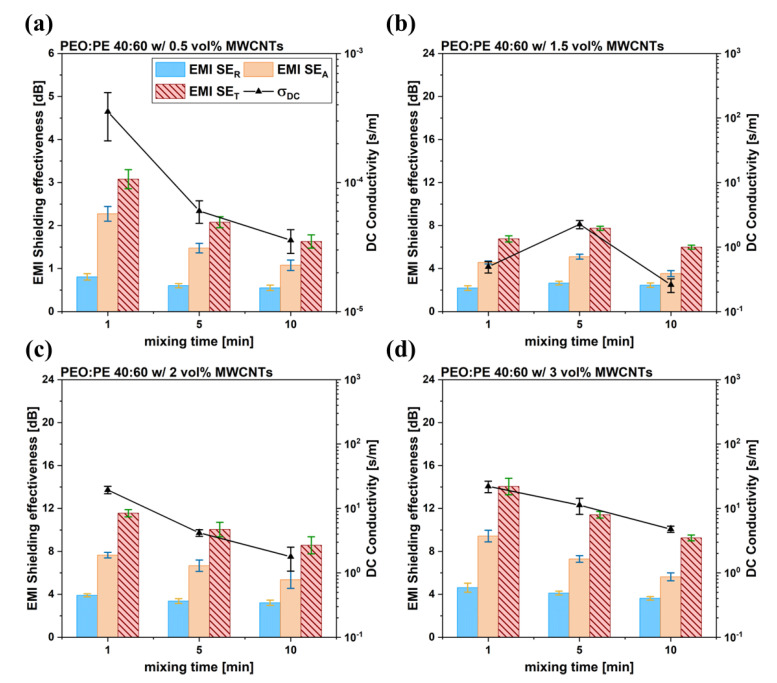
EMI SE values and DC electrical conductivity data for PEO/PE 40:60 blends containing MWCNTs at concentrations of (**a**) 0.5 vol%, (**b**) 1.5 vol%, (**c**) 2 vol%, and (**d**) 3 vol%.

Figure 4 shows the effect of mixing time and MWCNT concentration on the final observed DC electrical conductivity (σ_DC_) and EMI SE of PEO/PE 40:60 blends containing MWCNTs. σ_DC_ and EMI SE values for PEO and PE nanocomposites containing 3 vol% MWNCTs were also prepared to serve as a baseline, and the results can be found in Figure S6 of the Supplemental Materials. PEO/PE 40:60 blends at all MWCNT concentrations (Figure 4a,c,d) showed a decrease in the values of EMI SE and σ_DC_ with increasing mixing time. The overall decreasing trend can likely be attributed to the migration of MWCNTs from the PE phase into PEO, resulting in a breakdown of the conductive network. With MWCNTs localizing in both phases, double percolation no longer exists, i.e., because of the dilution of MWCNT concentration in PE phase, there is a reduction in the extent of MWCNT network formation. The high initial electrical properties can likely be attributed to MWCNTs forming a percolated network within PE exclusively, because of the firs mixing step before PEO was added. The most significant mechanism of EMI attenuation in all prepared 40:60 samples was absorption, which is common for PBNs [7]. PBNs tend to absorb incident EMI because the relatively miniscule quantity of MWCNTs at the surface of the samples allows EM waves to easily penetrate the bulk of the sample. Once within the sample, the electric and magnetic fields generated by the incident EM wave interact with the electric and magnetic dipoles within the embedded MWCNTs, leading to dissipation through ohmic losses. 

**Figure 5:**
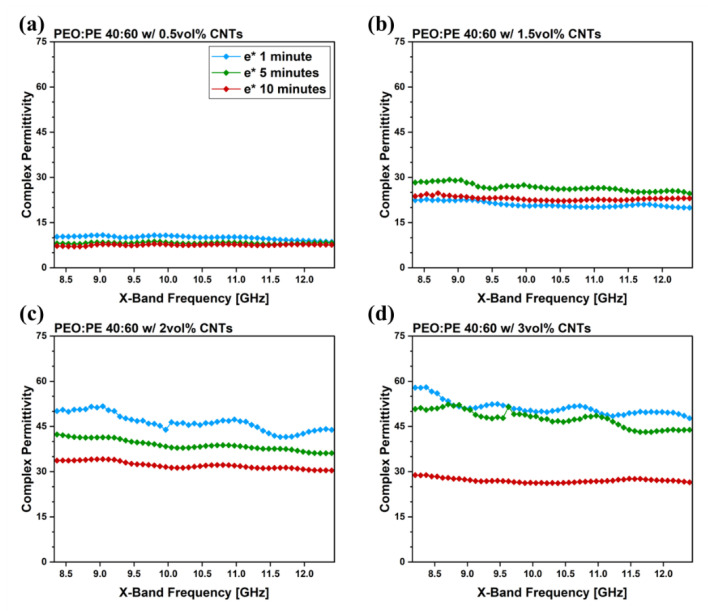
Complex permittivity data within the X-band for PEO/PE 40:60 blends containing MWCNTs at concentrations of (**a**) 0.5 vol%, (**b**) 1.5 vol%, (**c**) 2 vol%, and (**d**) 3 vol%.

The dielectric properties of the PEO/PE blends made in this work can also be studied. Complex electrical permittivity is typically described as:

$$\varepsilon^{*}=\;\varepsilon^{\prime }+{i\varepsilon }^{\prime \prime }\;\mathrm{or}\;\varepsilon^{*}=\sqrt{{\left(\;\varepsilon^{\prime }\right)}^{2}+{\left(\varepsilon^{\prime \prime }\right)}^{2}}$$

where $\;\varepsilon^{\prime }$ is the real permittivity and $\;\varepsilon^{\prime \prime }$ is the imaginary permittivity. The real permittivity of a material describes its ability to store electrical energy when subjected to an electric field, and the imaginary permittivity describes its ability to dissipate energy. In the case of PBN systems containing MWCNTs, $\;\varepsilon^{\prime }$ is caused by interfacial polarization between the polymer and MWCNTs, and $\;\varepsilon^{\prime \prime }$ is caused by charges dissipating through interconnected MWCNT networks [7]. Plots of $\;\varepsilon^{\prime }$ and $\;\varepsilon^{\prime \prime }$ for PEO/PE blends containing MWCNTs can be found in Figure S5 of the Supplementary Materials. Additionally, real and complex permittivity plots of PEO and PE containing 3 vol% MWCNTs can be found in Figure S7 of the Supplementary Materials. Figure 5 shows the $\varepsilon^{*}$ data for all prepared PEO/PE samples. When studying electrical permittivity in terms of $\varepsilon^{*}$, the similarities to the EMI SE and σ_DC_ values seen in Figure 4 become apparent. Looking at the $\varepsilon^{*}$ data in Figure 5, all curves closely match the results seen in Figure 4.

### 3.4. Rheological Properties: Linear Frequency Sweeps and Strain Sweeps

**Figure 6:**
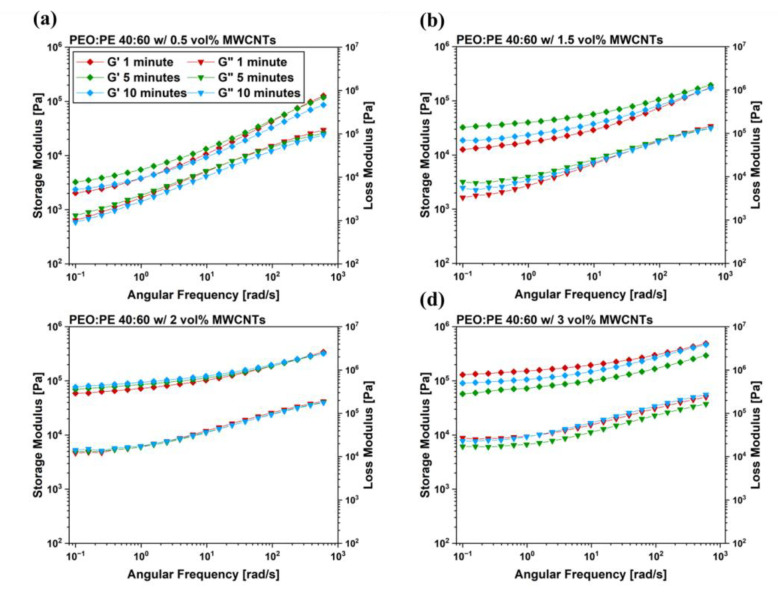
Frequency sweep results for PEO/PE 40:60 blends containing MWCNTs at concentrations of (**a**) 0.5 vol%, (**b**) 1.5 vol%, (**c**) 2 vol%, and (**d**) 3 vol%.

Frequency sweeps were performed on the mixed PBNs at a constant strain of 0.1% to ensure the samples were being studied in the linear viscoelastic (LVE) region, reducing the risk of disrupting the MWCNT networks formed within the PEO/PE blend samples. The results of the frequency sweeps are plotted in Figure 6. In addition to the rheology curves for PEO/PE blends plotted in Figure 6, frequency sweep plots for PEO and PE containing 3 vol% MWCNTs were also prepared to serve as a baseline and can be found in Figure S8 of Supplemental Materials. Frequency sweeps of pure PEO and PE, as well as their blends are available in Figure S3 and S4 of the Supplementary Materials. At MWCNT concentrations below the percolation threshold (0.5 and 1.5 vol% MWCNTs, shown in Figure 6a,b), the peak in viscoelastic moduli for low angular frequencies appears at 5 min of mixing. Previous works studying the rheological properties of MWCNT-filled polymer nanocomposites have suggested that a higher plateau of the storage and loss modulus (G’ and G”) at low angular frequencies suggests a more pronounced network structure of MWCNTs within the PBN system. Since MWCNTs are not effectively percolating within these systems, the peak in rheological properties at 5 min is likely due to MWCNTs dispersing into both the PEO and PE phase.

In the case of PEO/PE blend nanocomposites above the percolation threshold (2 vol% and 3 vol% MWCNTs, shown in Figure 6c,d), the trend in G’ and G’’ is not as consistent. This is likely due to competing effects between the disruption of the pre-existing MWCNT network within the PE phase, and the migration of MWCNTs into the PEO phase. For the system containing 2 vol% MWCNTs (Figure 6c), G’ and G’’ rise slightly with increasing mixing time, suggesting that the increase in properties caused by MWCNTs migrating into PEO outweighs the loss in properties caused by the disruption of the initial MWCNT network within PE. For 3 vol% MWCNT nanocomposites (Figure 6d), G’ and G’’ decrease over time. The loss in properties caused by the disruption of the highly robust MWCNT network initially present in PE has more significance than the increase in properties resulting from dispersing MWCNTs uniformly into PEO. 

**Figure 7:**
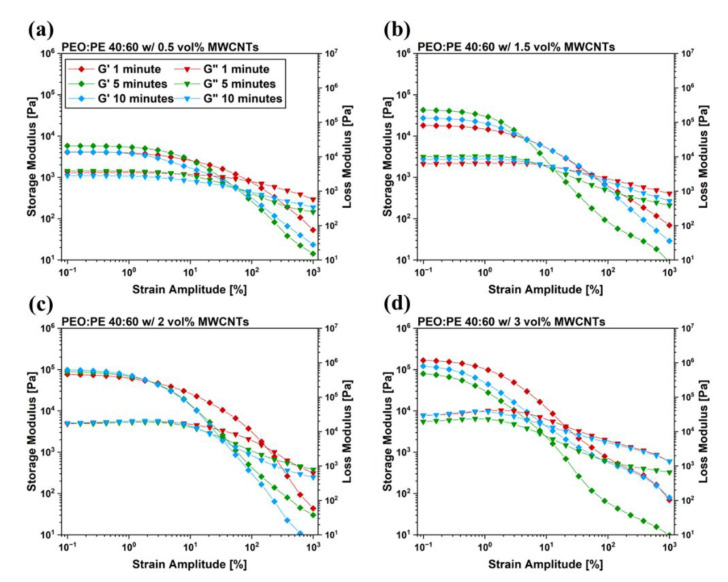
Strain sweep results for PEO/PE 40:60 blends containing MWCNTs at concentrations of (**a**) 0.5 vol%, (**b**) 1.5 vol%, (**c**) 2 vol%, and (**d**) 3 vol%.

Linear frequency sweeps were followed-up with strain sweeps to link the rupture of the microstructures within the blend systems to the extent of MWCNTs networks formed therein. Figure 7 shows the relationship between the strain amplitude and the observed rheological properties of the prepared 40:60 blends at varying MWCNT concentrations and mixing times. In addition to the strain sweep data prepared for the 40:60 blends, plots for PEO and PE containing 3 vol% were also prepared to serve as a baseline, which are plotted in Figure S8 of the Supplementary Materials. Furthermore, strain sweeps of pure PEO and PE, as well as their blends can be found in Figures S3 and S4 of the Supplementary Materials. As strain amplitude within the test exceeds a critical value, G’ values drop rapidly, and the samples exhibit a crossover point (i.e., the strain amplitude at which the storage modulus becomes larger than the loss modulus) [37]. This decrease in G’ is typically associated with the destruction of the existing MWCNT network [38]. Consequently, crossover points occurring at higher strain amplitudes are indicative of a more substantial MWCNT network within the system. The change in crossover points observed with increasing mixing time closely follows the trends observed in the EMI SE and σ_DC_ values seen in Figure 4. 40:60 blends with 0.5 vol%, 2 vol% and 3 vol% MWCNTs (Figure 7a,c,d) all show the most delayed crossover point occurring at 1 min of mixing, due to the robust MWCNT network present with PE at this time.

### 3.5. Compatibilization Effects—Changes in Morphological, Electrical, and Rheological Properties with the Addition of PEMA Compatibilizer

Studies into the effect of MWCNT concentration and mixing time on the morphological, electrical, and rheological properties of PEO/PE blends indicated substantial migration of MWCNTs from the PE phase into the PEO phase over time, as suggested by SEM and electrical conductivity/EMI SE measurements. PEO has a significantly lower viscosity than PE, which likely played a role in the migration of MWCNTs from PE to PEO. A PEMA compatibilizer was introduced to improve the interfacial adhesion between PE and PEO and to arrest the migration of MWCNTs at the blend interface. By forming crosslinks between the maleic anhydride groups of the PEMA molecules and the hydroxyl end groups of the PEO molecules, a more rigid interface, with superior adhesion can be created [39,40]. This interface serves to trap the MWCNTs as they migrate into PEO, arresting their movement, and preserving the desirable electrical and rheological properties observed in the PEO/PE 40:60 blends at 1 min of mixing. 

**Figure 8:**
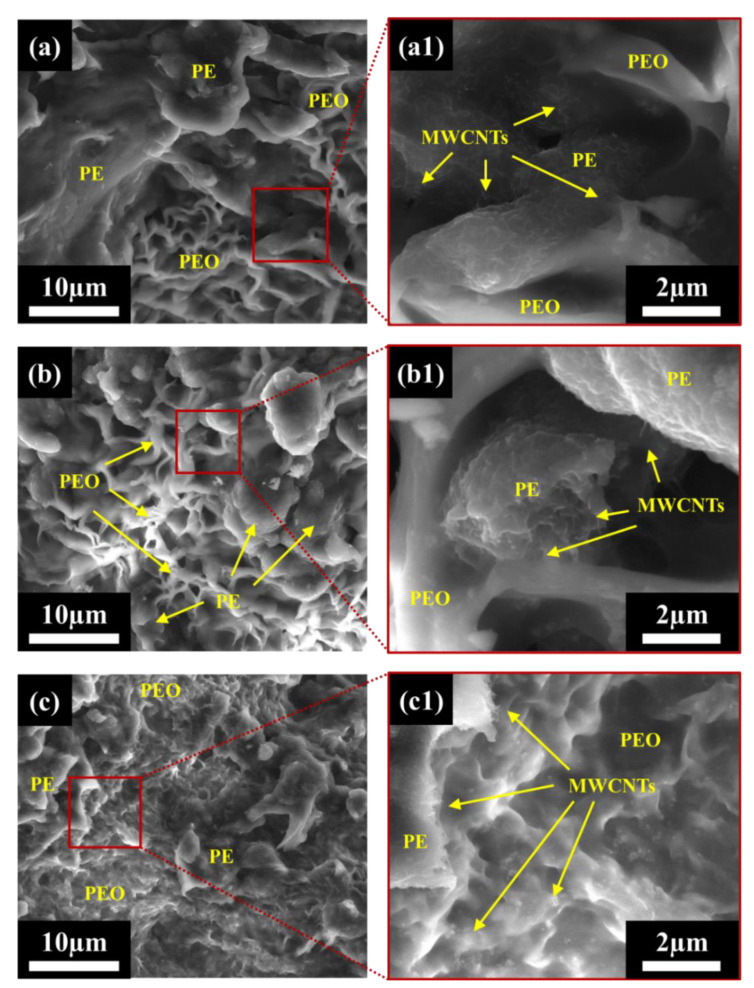
Low field detector (LFD) SEM images of PEO/PE/PEMA 40:54:6 with 3 vol% MWCNTs mixed for (**a**) 1 min, (**b**) 5 min, and (**c**) 10 min. Images (**a1**), (**b1**), and (**c1**) show PEO/PE/PEMA blend morphology at higher magnification.

SEM imaging was performed to study the blend morphology, and to identify the localization of MWCNTs within the prepared samples. Figure 8 shows SEM images of 40:54:6 blend samples containing 3 vol% MWCNTs. At 1 min of mixing (Figure 8a), PEO and PE phases can both be seen on the fracture surface, suggesting improved interfacial adhesion between the phases compared to the uncompatibilized blends. Furthermore, the PEO phases appear very rippled, which can also be indicative of compatibilization [41]. At higher magnification (Figure 8(a1)), numerous MWCNTs can clearly be seen along the PE surface, whereas MWCNTs appear near PEO only along the interface with PE. At 5 min of mixing (Figure 8b), the blend morphology appears far more refined, with smaller co-continuous domains of PE and PEO intermixing. At higher magnification (Figure 8(b1)), MWCNTs can once again be seen coating the PE surface and MWCNTs are visible along the interface with the PEO phase, which aligns with the high electrical and rheological properties seen at this point. At 10 min of mixing (Figure 8c), the PEO/PE blend morphology appears as before, suggesting that the limit of droplet breakup has been reached (coalescence is greatly inhibited by the presence of PEMA compatibilizer). At higher magnification (Figure 8(c1)), MWCNTs can be seen clearly in both PE and in PEO, suggesting that the migration of MWCNTs into PEO is extensive. This explains the greatly diminished electrical properties seen at 10 min of mixing, when compared to 5 min of mixing, since MWCNTs is now spread over both polymer phases.

**Figure 9:**
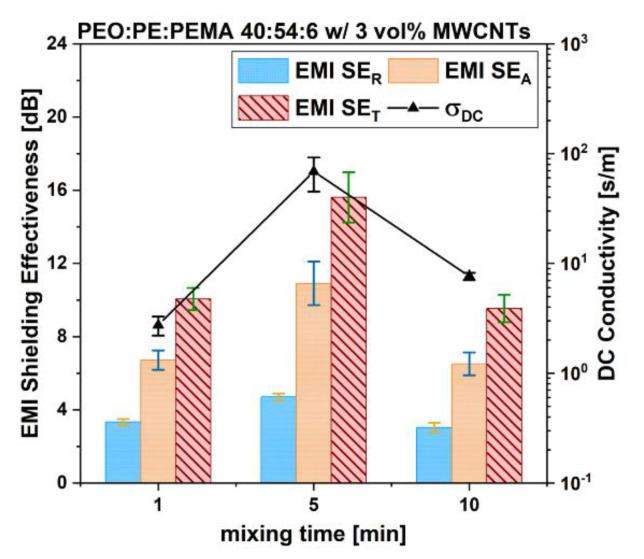
Low field detector (LFD) SEM images of PEO/PE/PEMA 40:54:6 with 3 vol% MWCNTs mixed for 1 min, 5 min, and 10 min.

Figure 9 shows the EMI SE and σDC values for PEO/PE/PEMA 40:54:6 blends containing 3 vol% MWCNTs. Because PEMA is more polar than PE, MWCNT may tend to move near the PEMA molecules. The electrical properties at 1 min of mixing are quite low, possibly due to MWCNTs concentrating in and around the PEMA phase within PE at the onset of mixing, leading to a loss of continuity in the MWCNT network. At 5 min of mixing however, there is a huge rise in both EMI SE and σDC. These values are even higher than the 40:60 blends with 3 vol% MWCNTs at 1 min mixing (15.6 dB and 68.7 S/m compared to 14.1 dB and 22.1 S/m), marking a significant improvement in the MWCNT network for the compatibilized blend. This improvement is likely due to MWCNTs being concentrated at the interface of the blend at 5 min, as evidenced by SEM images. The subsequent drop in properties at 10 min of mixing is likely due to MWCNTs finally crossing into PEO.

**Figure 10:**
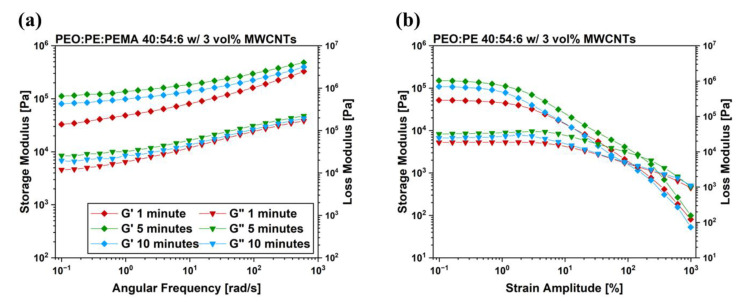
(**a**) Frequency sweep and (**b**) strain sweep results for PEO/PE 40:54:6 blends containing 3 vol% MWCNTs.

Figure 10 shows linear frequency and strain sweeps for 40:54:6 blends containing 3 vol% MWCNTs. As expected, 1 min of mixing yielded the lowest G’ and G’’ values at low angular frequencies (Figure 10a), likely due to no MWCNTs being present in PEO, and an inability of MWCNTs to form networks within the PE phase. At 5 min of mixing, the viscoelastic moduli rise sharply. This is due to the high degree of MWCNTs networks present within the blend, likely concentrated along the PEO/PE interface at this point. Furthermore, the critical strain in Figure 10b for 40:54:6 blends mixed for 5 min occurs at a substantially higher strain than at other times, suggesting a robust MWCNT network at this point. Although 1 min and 10 min samples yielded similar electrical properties, the values of G’ and G’’ are substantially higher at 10 min than at 1 min. This difference between the electrical and rheological properties is due to MWCNTs being more uniformly dispersed throughout both PE and PEO phases at 10 min, whereas at 1 min, MWCNTs are confined to the PE phase.

## 4. Discussion

### 4.1. Characteristics of MWCNT Migration in PEO/PE Blends

SEM imaging confirmed that more MWCNT phase migration from PE into PEO occurs with increasing mixing time. Figure 2 and Figure 3 show MWCNTs in the PE phase at all mixing times, with MWCNTs only being visible near the PEO/PE interface or within PEO at higher mixing times. The speed and extent to which this occurs also to rise with increasing MWCNT concentration. The faster migration of MWCNTs at 3 vol% is because there is a higher probability of MWCNTs encountering the PEO/PE interface during mixing at higher concentrations, and consequently MWCNT cross the interface and enter PEO.

The migration of MWCNTs into PEO is also supported by electrical properties of the PBNs with increasing mixing time (Figure 4 and Figure 5). At 1 min of mixing, MWCNTs are still within the PE phase, and form efficient interconnected networks due to the double percolation phenomenon. As the mixing time is increased, many MWCNTs reach the PEO/PE interface and migrate into the PEO phase, eventually becoming uniformly dispersed throughout the blend system. This leads to loss of double percolation, and a corresponding reduction in conductive properties. The rheological properties (Figure 6 and Figure 7) are similarly impacted. There are competing effects between having a uniform dispersion of MWCNTs throughout the blend system and having interconnected networks of MWCNTs that are entangled with one another. Below the percolation threshold (e.g., 0.5 vol% and 1.5 vol% MWCNTs), MWCNTs do not form networks or entangling with each other, therefore the improvement in rheological properties only occurs with MWCNTs dispersing uniformly throughout the blend. At MWCNT concentrations above the percolation threshold, the rheological properties at low mixing times may be higher due to the presence of entangled MWCNT networks. As the mixing time is increased, these MWCNT networks are disrupted as MWCNTs migrate into PEO and disperse throughout the blend. At 2 vol% (Figure 6c or Figure 7c), there is little change in properties over time because as the MWCNT network is destroyed, there is a gain in properties due to more uniform MWCNT dispersion; that is, the two effects cancel each other. In the case of 3 vol% (Figure 6d or Figure 7d), the pre-existing MWCNT network is far more significant, and its rupture over time leads to a decrease in properties with time.

The incorporation of PEMA compatibilizer slowed the migration of MWCNTs, and there was a delayed onset of peak electrical and rheological properties, which occurred at 5 min rather than 1 min of mixing for the uncompatibilized blend (Figure 9). SEM images showed that the blend morphology became finer over time, compared to uncompatibilized blends, suggesting that PEMA reached the PEO/PE interface, and inhibited the coalescence of the PEO phase. Thus, it is important to note that the addition of MWCNT may not only change the properties but also the type of morphology formed. However, the drop in electrical properties at 10 min of mixing, suggests that MWCNTs are ultimately able to cross the reinforced interface, despite the crosslinking of PE and PEO chains.

### 4.2. Predicting MWCNT Migration in Immiscible Polymer Blends

Based on our previous work studying the phase migration of MWCNTs in PVDF/PE blends, and the present work studying the migration of MWCNTs in PEO/PE blends, it is clear that there are several competing effects governing the final location of MWCNTs within polymer blend nanocomposites. In the case of the PVDF/PE system presented in our previous work [29], the high viscosity of PVDF (the phase with higher thermodynamic affinity for MWCNTs) helped to entrap MWCNTs at the blend interface. Although some individual MWCNTs were able to penetrate the PVDF/PE interface, and fully migrate into PEO, the majority of MWCNT agglomerates remained trapped on the PE side of the PVDF/PE interface. A modified “Slim-Fast Mechanism” originally presented by Göldel et al. [30] was proposed to explain the impact of MWCNT geometry on the ability of MWCNTs to migrate across the PVDF/PE interface; i.e., lone, straight MWCNTs penetrate the interface easily while MWCNT agglomerates do not and become locked at the interface. As MWCNT agglomerates jam at the PVDF/PE interface, they act as barriers for subsequent MWCNTs, leading to a cascading effect of most MWCNT become trapped at the interface, regardless of their geometry. In contrast, for the PEO/PE blend system studied in the present work, MWCNTs penetrate the PEO/PE interface and fully migrate into the low viscosity PEO and the migration continues as mixing time is increased. 

The differences in outcomes of MWCNT migration can be explained by looking at the surface energy data (Table 2) and the complex viscosity data (Table 3). Although the values of wettability are similar for the PVDF/PE and PEO/PE systems, the surface energies between individual component pairs varies significantly. In the case of the PVDF/PE system, the interfacial energies between either polymer and MWCNT is higher than the interfacial energies between the two polymers. This suggests that it is energetically unfavorable for incoming MWCNTs to penetrate the PVDF/PE interface, so MWCNTs failed to migrate from PE into PVDF, despite having a higher thermodynamic affinity for PVDF. In contrast, for the PEO/PE system, PEO has a higher thermodynamic affinity for MWCNT than PEO has for PE (i.e., σ_A/MWCNT_ < σ_A/B_). This means that it is energetically favorable for MWCNTs to migrate fully into PEO.

The complex viscosity data for the PVDF/PE blends shows that PVDF (i.e., the destination phase) had a significantly higher viscosity than PE throughout mixing, suggesting that the migration of MWCNTs would be kinetically hindered once they reached the PVDF side of the PVDF/PE interface. In contrast, the complex viscosity data for the PEO/PE blends show that PEO has a significantly lower viscosity than PE, which will facilitate the complete migration of MWCNTs once MWCNTs reach and penetrate the PEO/PE interface during mixing. While the same grade of PE is used as the initial phase in both systems, the viscosity of PE in the PEO/PE system is higher due to the lower processing temperature. Based on the literature, it is expected that this higher viscosity would impede the movement of MWCNTs towards the interface, but this does not appear to be the case since many MWCNTs rapidly migrated into PEO. This suggests that the viscosity of the pre-localized phase does not play as large of a role as the viscosity of the destination phase or of the specific interfacial surface energies between component pairs of the blend systems (e.g., σ_A/B_, σ_A/MWCNT_, etc.). When selecting a polymer blend in which interfacial localization of nanofillers is desired, it is crucial to select a blend in which the destination phase has a higher viscosity than the pre-localized phase. It is also important to select a blend not only based on the theoretical wettability values obtained via Young’s equation [21] but also based on the interfacial surface energies of the individual component pairs. For example, if the two polymer phases have a higher affinity for each other than with the nanofiller, it will be energetically unfavorable for the nanofiller to penetrate the interface.

## 5. Conclusions

The effects of MWCNT concentration, mixing time, and compatibilizer addition on the migration of MWCNTs from the polyethylene (PE) phase to a polyethylene oxide (PEO) phase of a 60:40 PEO/PE blend and the subsequent impact on electrical properties and rheological properties were investigated. Two-step mixing was used to pre-localize MWCNTs in the less thermodynamically favored PE phase and observe their migration into the thermodynamically favored PEO phase. SEM micrographs showed that MWCNTs migrate into the PEO phase as the mixing time increases at all concentrations of MWCNTs studied. This migration is also supported by EMI SE and DC conductivity measurements, which showed significant reductions in electrical properties over time, suggesting a disruption of conductive networks as MWCNTs migrate into PEO.

PEO/PE 40:60 samples containing 3 vol% MWCNTs showed a high conductivity of 22.1 S/m, respectively, suggesting effective MWCNT networks were present at the onset of mixing. To arrest the migration of MWCNTs into PEO, a PE-graft-maleic anhydride (PEMA) compatibilizer was added to the PEO/PE blend. SEM images confirm an improvement in the formation of MWCNT networks along the PEO/PE interface at 5 min of mixing for the compabilized PBN. Furthermore, major improvements in electrical conductivity (68.7 S/m) were observed. Comparisons to the PVDF/PE system studied in our previous work suggest that the viscosity of the destination phase, as well as the interfacial surface energies of the blend components play significant roles in determining whether MWCNTs will successfully migrate across polymer/polymer interfaces or whether they will become trapped at the interface. The migration behavior was shown to significantly influence the electrical and rheological properties of PBNs.

## Figures and Tables

**Table 1:** Surface Energies and Wettability of MWCNTs within PEO and PE at 150 °C.

Parameter	Geometric Mean ^1^	Harmonic Mean ^2^
σ_PEO/PE_ [mJ/m^2^]	9.31	9.39
σ_PEO/MWCNT_ [mJ/m^2^]	5.80	9.32
σ_MWCNT/PE_ [mJ/m^2^]	27.65	28.39
Wettability	−2.35	−2.03

^1,2^ Wettability parameters were calculated using data from Wu [35] and Owens [36].

**Table 2:** Surface Energy and Wettability Data for PVDF/PE and PEO/PE Blend Systems Containing MWCNTs (calculated based on geometric mean) [29].

**PBN System** **[A/B]**	Temperature [°C]	$\sigma_{A/B}$ [mJ/m^2^]	$\sigma_{A/MWCNT}$ [mJ/m^2^]	$\sigma_{B/MWCNT}$ [mJ/m^2^]	ω
PVDF/PE	200	7.00	11.74	27.18	−2.21
PEO/PE ^1^	150	9.31	5.80	27.65	−2.35

^1^ Current work.

**Table 3:** Complex Viscosity Data of PVDF/PE and PEO/PE Systems [29].

PBN System [A/B]	Temperature [°C]	$\mu_{A}$ [Paˑs]	$\mu_{B}$ [Paˑs]	$\frac{\mu_{A}}{\mu_{B}}$
PVDF/PE	200	3.14**ˑ**10^2^	4.70**ˑ**10^2^	0.67
PEO/PE ^1^	150	4.66**ˑ**10^2^	1.62**ˑ**10^2^	2.88

^1^ Current work.

## Data Availability

The data presented in this work are available upon request from the corresponding author.

## Supplementary Materials

The following supporting materials can be downloaded at https://www.mdpi.com/article/10.3390/nano12213772/s1. Figure S1. LM images for PEO/PE 40:60 blends containing MWCNTs, including—blends containing 0.5vol% MWCNTs mixed for (a) 1min, (b) 5min, and (c) 10min; blends containing 1.5vol% MWCNTs mixed for (d) 1 min, (e) I.5min, and (f) 10min; blends containing 2vol% MWCNTs mixed for (g) 1min, (h) 5min, and (i) 10min; blends containing 3vol% MWCNTs mixed for (j) 1min, (k) 5min, and (l) 10min. PE composites containing MWCNTs at concentrations of (m) 0.5vol%, (n) 1.5vol%, (o) 2vol%, and (p) 3vol% are also included; Figure S2. SEM images of neat (a) 40:60 PEO/PE blends, (b) 40:57:3 PEO/PE/PEMA blends, and (c) 40:54:6 PEO/PE/PEMA blends, all mixed for 10 minutes; Figure S3. (a) Frequency sweep data and (b) strain sweep data for pure PEO, PE and PEMA; Figure S4. (a) Frequency sweep data and (b) strain sweep data for PEO/PE 40:60 blends containing varying concentrations of PEMA compatibilizer; Figure S5. Real and imaginary permittivity data within the X-band for PEO/PE 40:60 blends containing MWCNTs at concentrations of (a) 0.5vol%, (b) 1.5vol%, (c) 2vol%, and (d) 3vol%; Figure S6. EMI SE and DC conductivity data for (a) PE and (b) PEO nanocomposites containing 3vol% MWCNTs; Figure S7. Electrical permittivity data across the X-band for PE and PEO nanocomposites containing 3vol% MWCNTs, including—(a) (b) real and imaginary permittivity, and (c) (d) complex permittivity; Figure S8. (a,b) Frequency sweep data and (c,d) strain sweep data for PE and PEO nanocomposite samples containing 3vol% MWCNTs;. Surface energy values for each component within the PVDF/PE system at 150 °C [29,30].
