# Supplementary material for: Carbon Nanotube Migration in Melt-Compounded PEO/PE Blends and Its Impact on Electrical and Rheological Properties
Source: Nanomaterials (Basel). 2022 Oct 26;12(21):3772. doi: 10.3390/nano12213772 (PMC9656623; doi:10.3390/nano12213772)
Supplement: Supplementary file 1 [file nanomaterials-12-03772-s001.zip › nanomaterials-1975888-supplementary.pdf]

Supplementary Materials

A.1 LM images for PEO/PE/MWCNTs blends

LM images (Figure S1) were taken of PEO/PE blends containing MWCNTs, to identify the localization of MWCNTs within the blend system as the MWCNT concentration and the mixing time is increased. PE/MWCNT composite is provided as a reference. The dark phase is PE, which is rich in MWCNTs at the beginning of mixing, whereas the PEO phase is far lighter. As the mixing time is increased, the surface area occupied by the PEO phase appears to decrease, likely due to MWCNT migrating in from the PE phase. The images on the right-hand side of Figure S1 are of PE/MWCNT blends, with the black spots being MWCNT agglomerates. Regardless of concentration, the size and frequency of MWCNT agglomerates is low, suggesting a high-quality dispersion of MWCNTs within the PE phase during mixing.

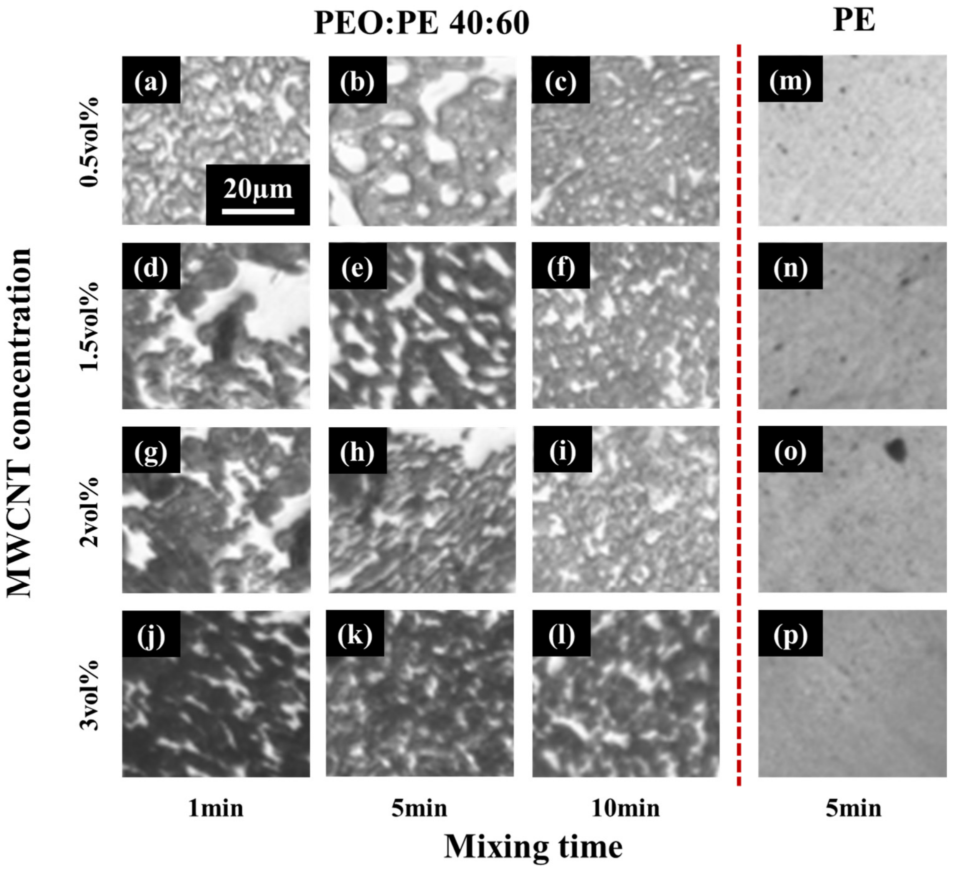

Figure S1. *LM images for PEO/PE 40:60 blends containing MWCNTs, including – blends containing 0.5vol% MWCNTs mixed for (a) 1min, (b) 5min, and (c) 10min; blends containing 1.5vol% MWCNTs mixed for (d) 1 min, (e) 1.5min, and (f) 10min; blends containing 2vol% MWCNTs mixed for (g) 1min, (h) 5min, and (i) 10min; blends containing 3vol% MWCNTs mixed for (j) 1min, (k) 5min, and (l) 10min. PE composites containing MWCNTs at concentrations of (m) 0.5vol%, (n) 1.5vol%, (o) 2vol%, and (p) 3vol% are also included.*

A.2 SEM images for pure PEO/PE blends and PEO/PE/PEMA blends

The geometric and harmonic means derived by Wu [29] can be used as follows:

$$\sigma_{12,\text{harmonic}} = \sigma_1 + \sigma_2 - 4\left[\frac{\sigma_1^d\sigma_2^d}{\sigma_1^d + \sigma_2^d} + \frac{\sigma_1^p\sigma_2^p}{\sigma_1^p + \sigma_2^p}\right] \text{ (eq. S1)}$$

$$\sigma_{12,\text{geometric}} = \sigma_1 + \sigma_2 - 4\left[\frac{\sigma_1^d\sigma_2^d}{\sigma_1^d + \sigma_2^d} + \frac{\sigma_1^p\sigma_2^p}{\sigma_1^p + \sigma_2^p}\right] \text{ (eq. S2)}$$

Where σ_i is the total surface energy of component i, σ_i^d is the dispersive surface free energy of component i, and σ_i^p represents the polar surface free energy of component i. The individual surface energies for each component are summarized in Table S1.

Table S1. Surface energy values for each component within the PVDF/PE system at 150°C [29, 30].

**Component**	**Total Surface Free Energy (mJ/m^2)**	**Dispersive Surface Free Energy (mJ/m^2)**	**Polar Surface Free Energy (mJ/m^2)**
PEO	42.9	30.9	12.0
PE	35.3	35.3	0.00
MWCNTs	45.3	18.4	26.9

A.3 SEM images for pure PEO/PE blends and PEO/PE/PEMA blends

Scattering parameters measured by the network analyzer were used to determine the EMI SE values for the prepared samples. $EMI\ SE_R$ and $EMI\ SE_A$ were calculated from scattering parameters as follows [7]:

$$R = \left|\frac{P_R}{P_I}\right| = |S_{11}|^2 = |S_{22}|^2 \text{ (eq. S3)}$$

$$T = \left|\frac{P_T}{P_I}\right| = |S_{12}|^2 = |S_{21}|^2 \text{ (eq. S4)}$$

$$\text{EMI SE}_R = 10 \cdot \log\left(\frac{1}{1-R}\right) \text{ (eq. S5)}$$

$$\text{EMI SE}_A = 10 \cdot \log\left(\frac{1-R}{T}\right) \text{ (eq. S6)}$$

Where R is the reflectance, T is the transmittance, P_R is the power of the reflected wave, P_I is the power of the incident wave and P_T is the power of the transmitted wave. Additionally, S_{11}, S_{12}, S_{21} and S_{22} are the forward reflection coefficient, forward transmission coefficient, reverse transmission coefficient and the reverse reflection coefficient, respectively.

A.4 SEM images for pure PEO/PE blends and PEO/PE/PEMA blends

SEM images (Figure S2) were taken of pure PEO/PE blends, as well as corresponding blends containing increasing concentrations of PEMA compatibilization, to establish the effect of the addition of PEMA compatibilizer on the final morphology of PEO/PE blends.

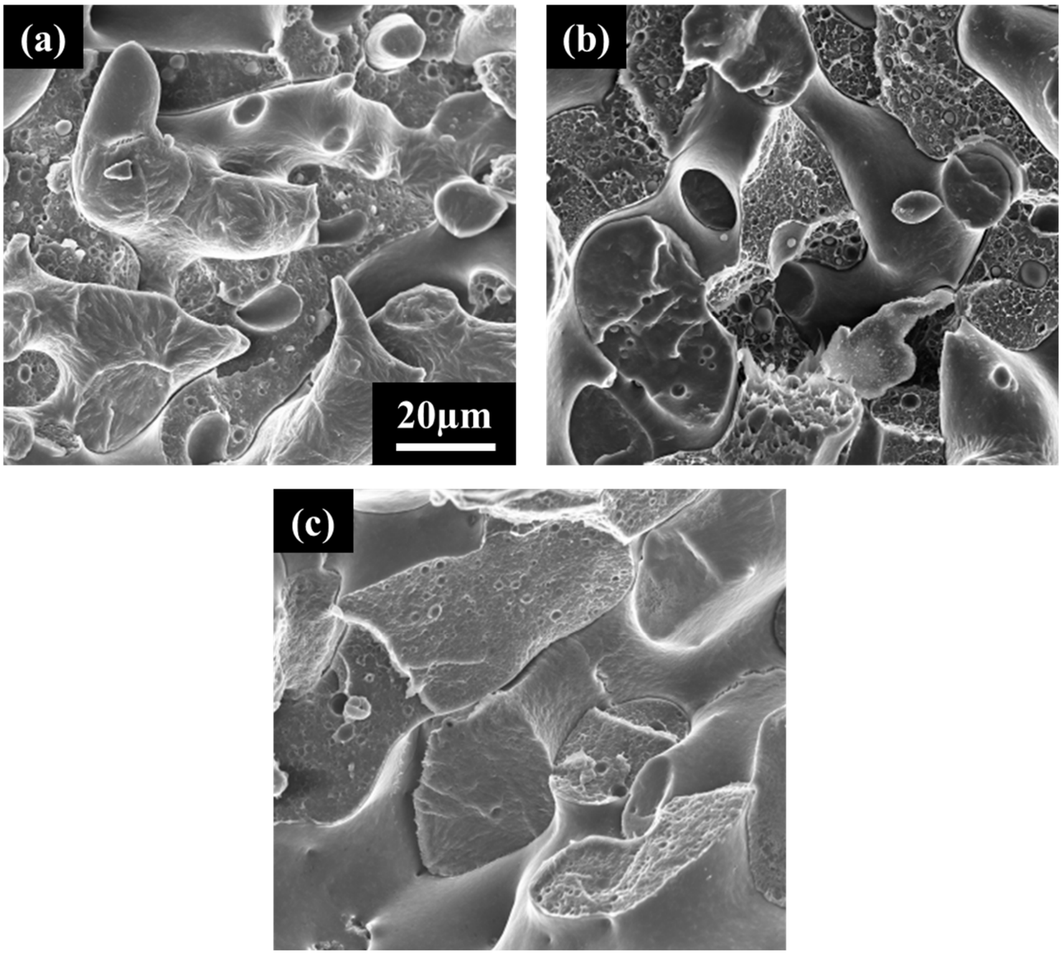

Figure S2. *SEM images of neat (a) 40:60 PEO/PE blends, (b) 40:57:3 PEO/PE/PEMA blends, and (c) 40:54:6 PEO/PE/PEMA blends, all mixed for 10 minutes.*

A.5 Frequency and strain sweep data for pure PEO, PE and PEMA

Frequency and strain sweeps were performed on the PEO, PE and PEMA prepared in this work. Frequency sweeps were performed at a constant strain of 0.1%, and strain sweeps were performed at a constant angular frequency of 1 rad/s. All testing was performed at a temperature of 150°C. The results of these tests are plotted in Figure S3 below.

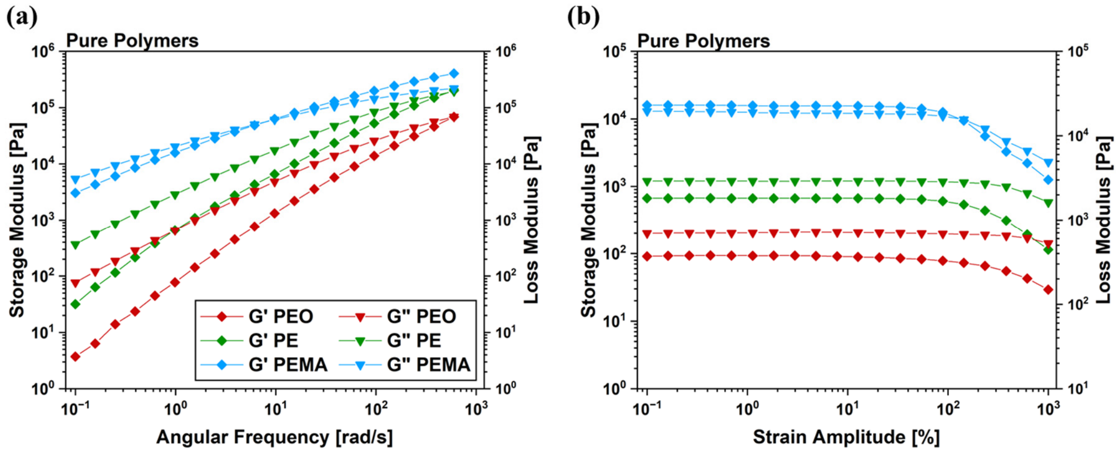

Figure S3. *(a) Frequency sweep data and (b) strain sweep data for pure PEO, PE and PEMA.*

A.6 Frequency and strain sweep data for pure PEO/PE blends and PEO/PE/PEMA blends

Frequency and strain sweeps were performed on the PEO/PE 40:60 blends containing 0vol%, 3vol%, and 6vol% of added PEMA compatibilizer, to demonstrate the compatibilization effect of adding PEMA. The results are plotted in Figure S4 below. Frequency sweep results in Figure S4(a) show increasing shouldering of both the storage and loss modulus at low frequencies with increasing PEMA content, suggesting the presence of a more robust structure within the blend. This structure is possibly due to reduced slippage between the PEO and PE phases, caused by improved adhesion from the PEMA compatibilizer. Strain sweep plots in Figure S4(b) show higher initial viscoelastic moduli, but more rapid decline with increasing strain, suggesting the destruction of the blend structure created from compatibilization with PEMA.

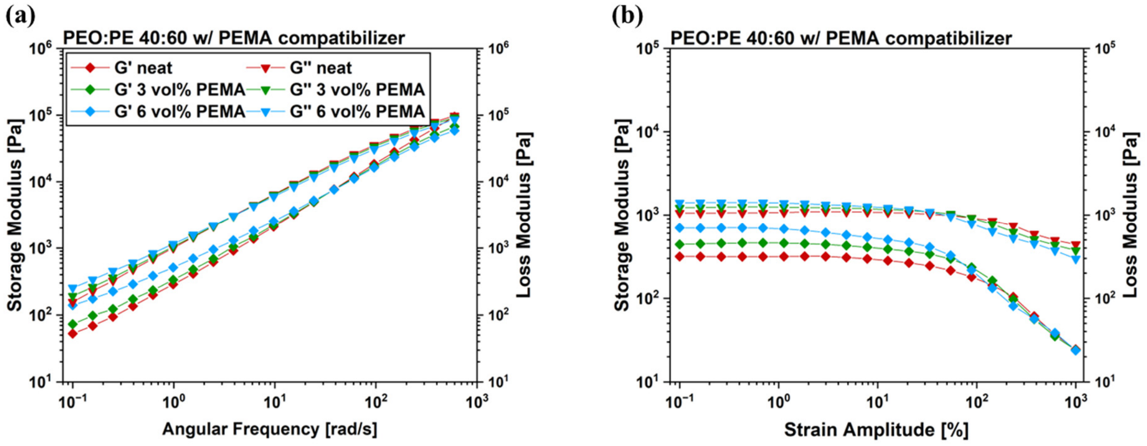

Figure S4. *(a) Frequency sweep data and (b) strain sweep data for PEO/PE 40:60 blends containing varying concentrations of PEMA compatibilizer.*

A.7 Real and imaginary permittivity data for PEO/PE blends containing MWCNTs

Figure S5 shows the ε' and ε'' values within the X-band for prepared 40:60 samples. As the concentration of MWCNTs is increased, both the values of ε' and ε'' increase, which is expected, since both parameters contribute to the EMI SE characteristics of the material. More importantly, the relative difference between the values of ε' and ε'' also increase in favor of ε'' with increasing MWCNT concentration, suggesting improvements in the conductive properties of the materials. In all cases, the permittivity values at 10min are the lowest, suggesting both worse charge storage and dissipation capabilities of the materials at high mixing times. This behavior is likely due to reduced MWCNT networks present within the blends at these times, as well as a more inefficient dispersion of MWCNTs within the blends at these times (i.e., MWCNTs may be concentrated near the PEO/PE interface, but unable to form conductive networks). Significant rises in the values of ε' and ε'' can be seen starting at 40:60 blends containing 2vol% MWCNTs (Figure S5(c)). At 1min, the values of ε'' even surpass ε', suggesting a system governing by charge dissipation rather than polarization. This change in behavior between samples containing 1.5vol% MWCNTs and those containing 2vol% MWCNTs is likely due to the onset of percolation within the system between these two points.

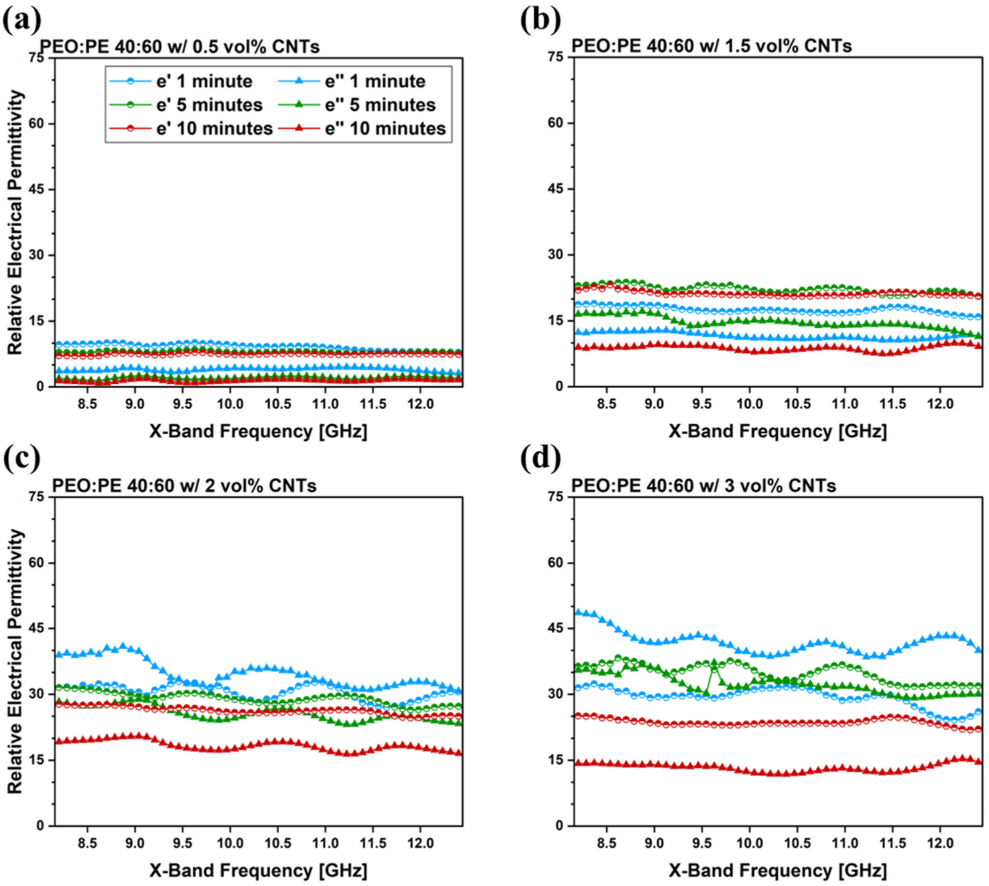

Figure S5. *Real and imaginary permittivity data within the X-band for PEO/PE 40:60 blends containing MWCNTs at concentrations of (a) 0.5vol%, (b) 1.5vol%, (c) 2vol%, and (d) 3vol%.*

A.8 Electrical/rheological properties of PEO and PE nanocomposites with 3vol% MWCNTs

In addition to preparing blends of PEO/PE, pure PEO and PE nanocomposite samples were prepared and characterized to serve as an additional baseline for performance. The electrical and rheological results are plotted below. All EMI shielding and permittivity measurements were performed across the X-band. Results show exceptionally high electrical properties for PEO nanocomposites, especially at 5 and 10min of mixing. Furthermore, the viscoelastic moduli at 5 and 10min of mixing are also exceptionally high. Both of these results suggest the formation of substantial MWCNT networks within PEO, likely due to the low viscosity of PEO and its high affinity for MWCNTs, compared to PE.
In contrast, PE nanocomposite samples show lower electrical properties, when compared to the PEO nanocomposite samples. This is likely due to PE's lower affinity for MWCNTs, and its substantially higher viscosity, both of which hinder the MWCNTs ability to disperse effectively within the matrix phase and form interconnected conductive networks. Interestingly, despite PE's inherently higher rheological compared to PEO, the prepared PEO and PE nanocomposites show very similar rheological properties. This is once again due to the more significant MWCNT structure being formed within PEO, leading the greater improvements in the rheological properties. Like their PEO/MWCNT counterparts, PE/MWCNT sample exhibit significantly worse electrical and rheological properties at 1min of mixing, and the greatest properties at 5min of mixing. This is likely due to MWCNTs not having adequate to disperse during 1min of mixing either system, while becoming optimally dispersed by 5min. The slight drop at 10min of mixing could be due to MWCNT scission as a results of shear forces exerted on them by the matrix phase.

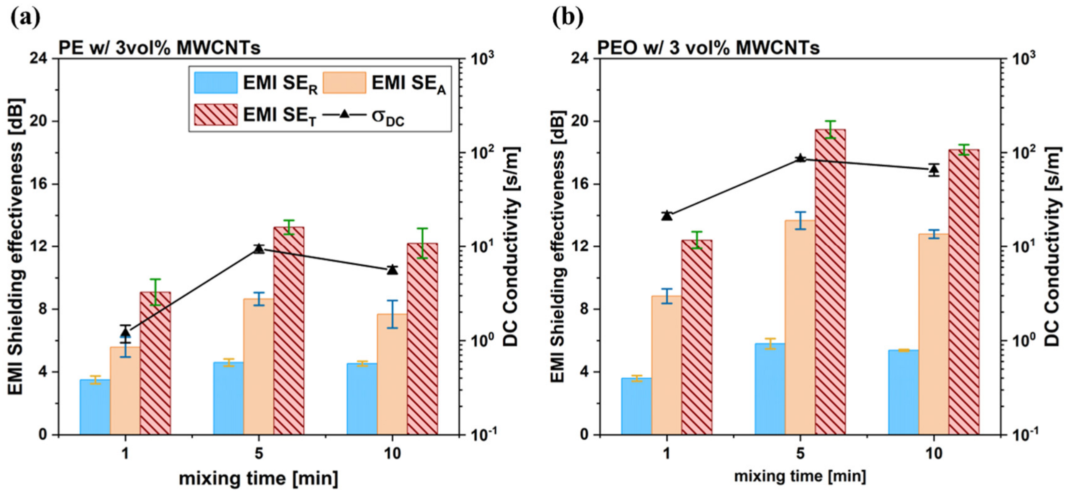

Figure S6. *EMI SE and DC conductivity data for (a) PE and (b) PEO nanocomposites containing 3vol% MWCNTs.*

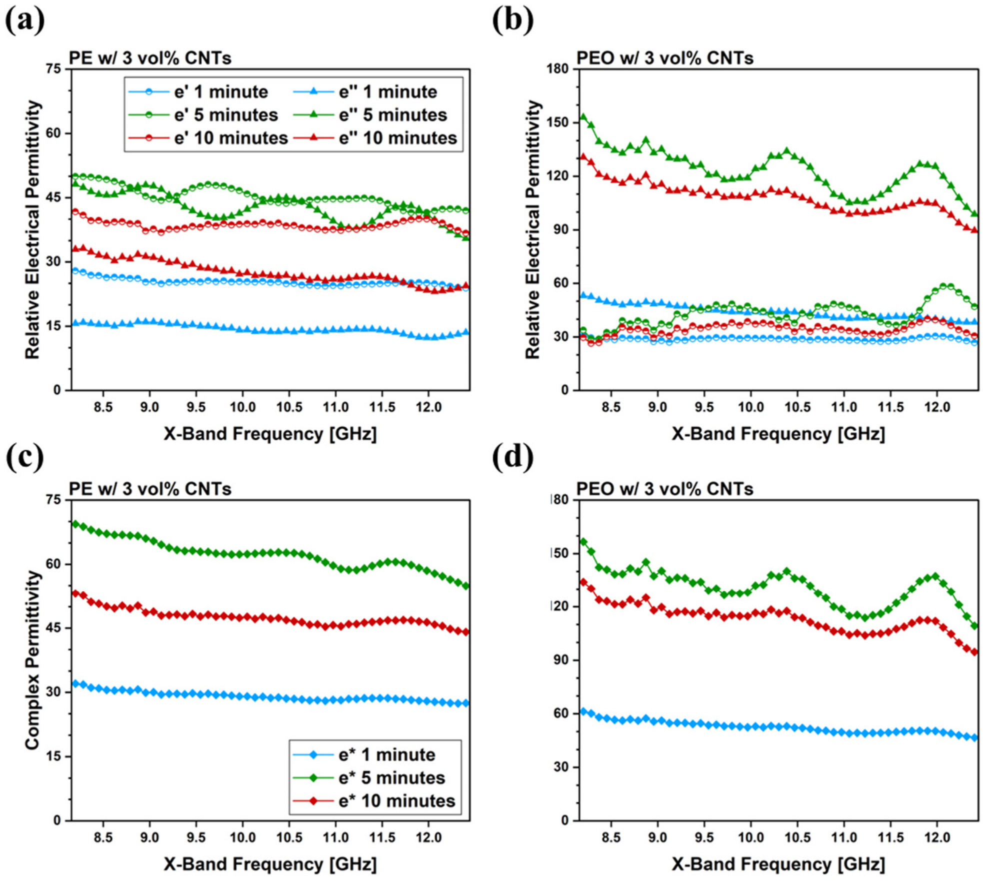

Figure S7. *Electrical permittivity data across the X-band for PE and PEO nanocomposites containing 3vol% MWCNTs, including – (a) (b) real and imaginary permittivity, and (c) (d) complex permittivity.*

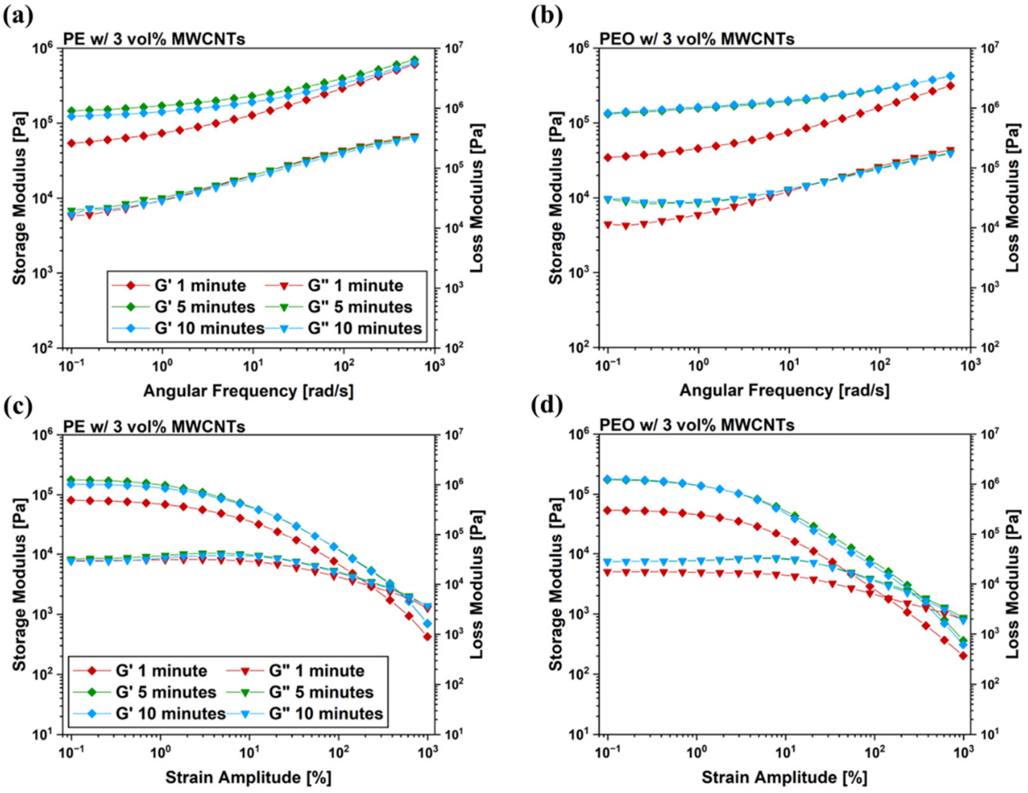

Figure S8. *(a, b) Frequency sweep data and (c, d) strain sweep data for PE and PEO nanocomposite samples containing 3vol% MWCNTs.*
